# 3D Graphene-Nanowire “Sandwich” Thermal Interface with Ultralow Resistance and Stiffness

**DOI:** 10.1021/acsnano.2c10525

**Published:** 2023-01-17

**Authors:** Lin Jing, Rui Cheng, Raghav Garg, Wei Gong, Inkyu Lee, Aaron Schmit, Tzahi Cohen-Karni, Xu Zhang, Sheng Shen

**Affiliations:** † Department of Mechanical Engineering, Carnegie Mellon University; Pittsburgh, Pennsylvania 15213, United States; ‡ Department of Materials Science and Engineering, Carnegie Mellon University; Pittsburgh, Pennsylvania 15213, United States; § Department of Mechanical Engineering, Massachusetts Institute of Technology; Cambridge, Massachusetts 02139, United States; ∥ Department of Electrical and Computer Engineering, Carnegie Mellon University; Pittsburgh, Pennsylvania 15213, United States

**Keywords:** thermal interface material, 3D “sandwich”, graphene−Cu nanowire, ultralow thermal resistance, high flexibility, high reliability

## Abstract

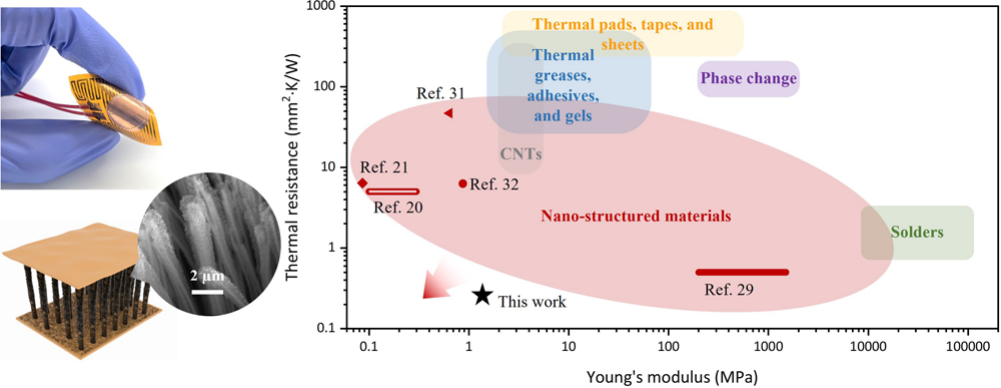

Despite the recent advancements of passive and active cooling solutions for electronics, interfaces between materials have generally become crucial barriers for thermal transport because of intrinsic material dissimilarity and surface roughness at interfaces. We demonstrate a 3D graphene-nanowire “sandwich” thermal interface that enables an ultralow thermal resistance of ∼0.24 mm^2^·K/W that is about 1 order of magnitude smaller than those of solders and several orders of magnitude lower than those of thermal greases, gels, and epoxies, as well as a low elastic and shear moduli of ∼1 MPa like polymers and foams. The flexible 3D “sandwich” exhibits excellent long-term reliability with >1000 cycles over a broad temperature range from −55 °C to 125 °C. This nanostructured thermal interface material can greatly benefit a variety of electronic systems and devices by allowing them to operate at lower temperatures or at the same temperature but with higher performance and higher power density.

Flexible electronics are transforming the way that we interact with our environments.^1−3^ They have generated exciting applications, including flexible LEDs^4^ and lasers^5^ for lighting and display, wearable sensors for communication,^6^ implantable electronics for monitoring health and imaging,^7^ and soft robotics.^8^ However, heat dissipation in flexible electronics has become a critical challenge and limited their performance and reliability because they are highly heterogeneous and involve diverse materials, including semiconductors, polymers, metals, and ceramics.^9^ Other than efficient heat transfer, heat removal in flexible electronics also requires highly flexible thermal solutions, such that they can be applied on curved and soft surfaces. In microelectronics, the escalating integration density in the last six decades has pushed forward the computing capabilities at the cost of rising heat dissipation across the device, circuit, and system levels.^10,11^ To date, the heat fluxes of high-power electronic devices and systems such as SiC high-electron-mobility transistors, solid-state lasers, and phased-array radars, can reach the order of 1 kW/cm^2^.^12−15^ Heat rejection technology has been playing a critical role in maintaining the Moore’s law scaling in the microelectronics industry.^16^ Despite the recent advancements of passive and active cooling solutions for electronics, interfaces between materials are still the crucial barriers for thermal transport because of the intrinsic material dissimilarity and surface roughness at interfaces. In many cases, thermal resistance at interfaces can constitute more than 50% of the total thermal resistance from an electronic device to a cooling fluid.^17,18^

Thermal interface materials (TIM) have been widely employed to mechanically joint and thermally bridge the interfaces. To enable a wide range of applications in flexible electronics and microelectronics, the “ideal” thermal interface materials must simultaneously possess high thermal conductivity for minimizing thermal resistance and high flexibility and compliance for adapting to soft and curved surfaces while accommodating the thermal stress derived from the mismatch of thermal expansion between two jointed materials. However, conventional thermal interface materials, such as solders, greases, gels, and epoxies, cannot satisfy such demanding and stringent technical criteria. Solders display high thermal conductivity but feature poor mechanical compliance.^19^ Thermal greases, epoxies and other polymer-based composites have high compliance but struggle with low thermal conductivity.^20,21^ Large-scale high-aspect-ratio nanostructures, including carbon nanotubes (CNTs),^22−26^ nanowires (NWs),^27−29^ graphene,^30−32^ nanosheets,^33^ and nanofibers,^34^ have offered a promising platform for fabricating thermally conductive and mechanically compliant thermal interface materials. Nevertheless, since these nanostructures are not intrinsically adhesive or solderable, physically bonding these nanostructures to a target substrate with a continuous interface and a low contact resistance remains an outstanding challenge. Here, we demonstrate a 3D nanostructured TIM on the basis of highly compliant and thermally conductive graphene-coated Cu nanowires (g-CuNWs) sandwiched between two Cu thin films. The whole structure is fully free-standing, soft, and flexible with a thickness of <40 μm and can be applied to a broad range of flexible and warped surfaces in electronics (Figure 1a). To the best of our knowledge, the 3D “sandwich” TIM shows superior thermal and mechanical properties beyond any existing thermal interface materials (Figure 1b).

**Figure 1:**
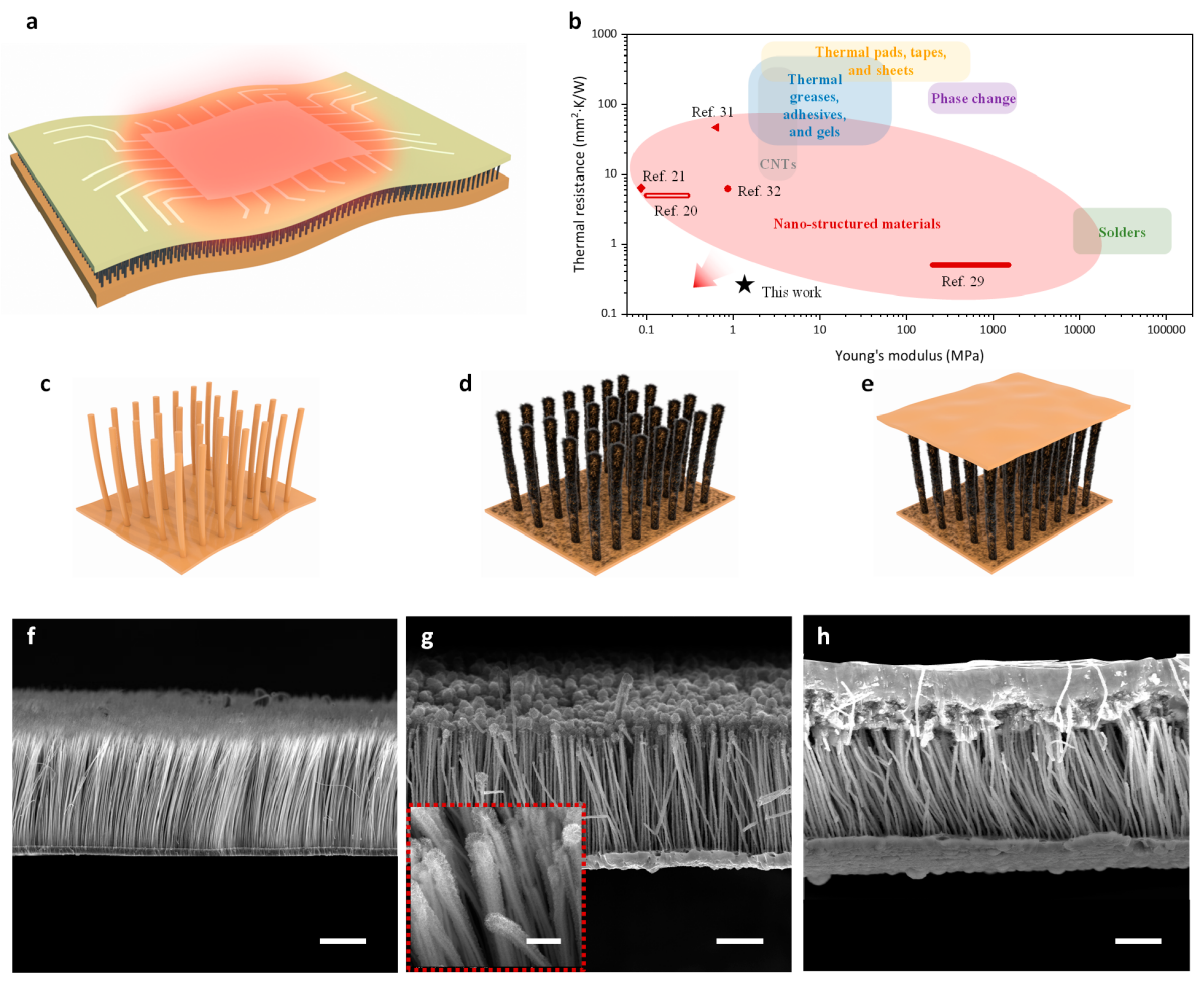
Flexible 3D graphene-nanowire “sandwich” TIM. (a) Schematic of the thermal management of flexible electronics with the 3D graphene-nanowire “sandwich” TIM. (b) Performance comparison of the 3D “sandwich” TIM with current state-of-the-art materials. (c,f) Schematic and SEM image of the vertically aligned Cu nanowire (CuNW) array as a scaffold for graphene growth. (d,g) Schematic and SEM image of the graphene-coated Cu nanowire (g-CuNW) array in which out-of-plane dense graphene flakes are conformally deposited on the CuNWs by PECVD. Inset of (g): Zoomed-in image of thick graphene flakes coated on CuNWs. (e,h) Schematic and SEM image of the 3D graphene-nanowire “sandwich” TIM consisting of compliant and thermally conductive g-CuNWs sandwiched between two Cu thin films. Scale bar in f, g, and h: 5 μm. Scale bar in the inset of g: 2 μm.

## Results and Discussion

**Figure 2:**
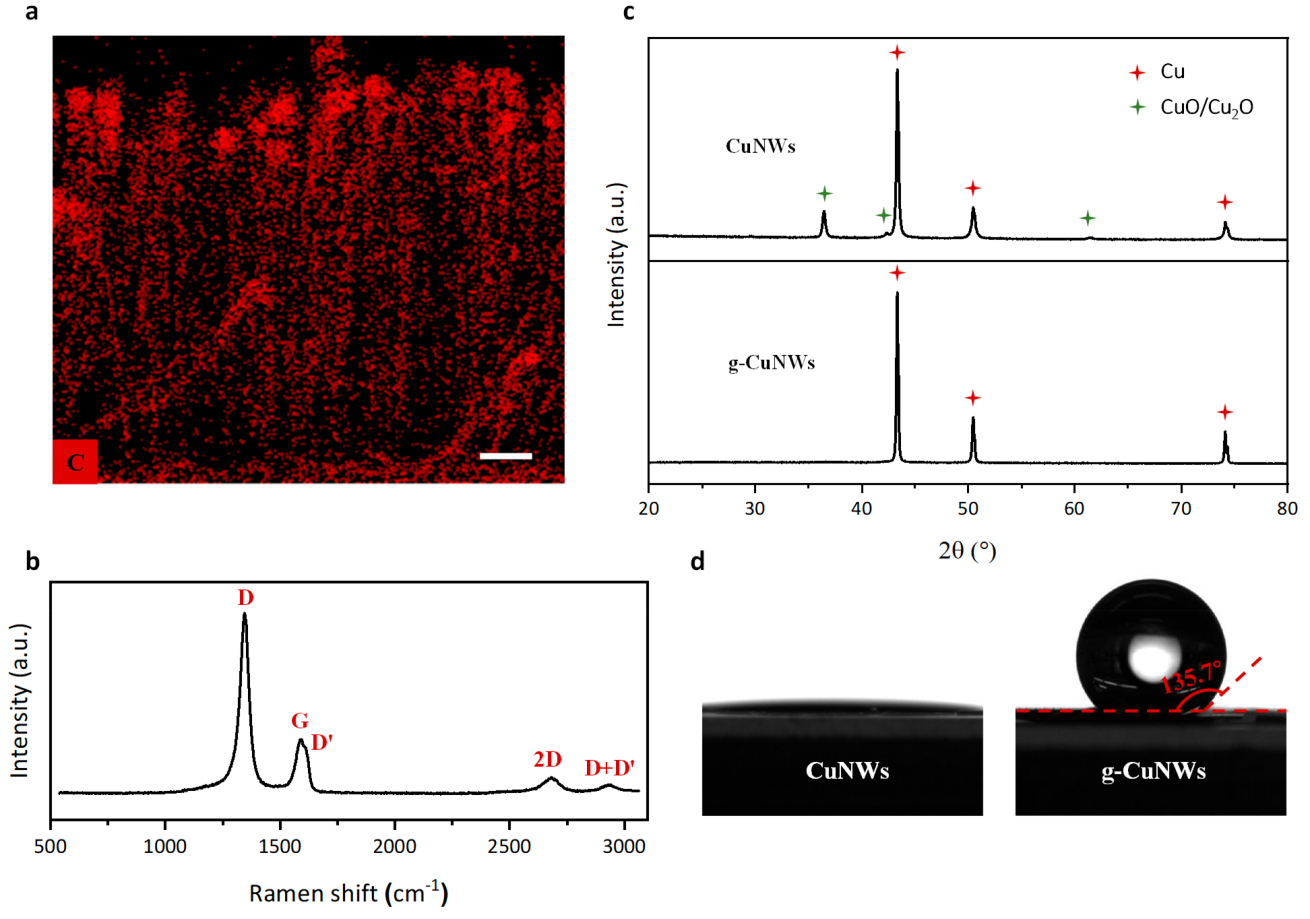
Material characterization of the CuNWs and g-CuNWs. (a) EDS mapping of the g-CuNWs, illustrating conformal carbon (red dots) coverage along the entirety of the CuNWs. Scale bar: 2 μm. (b) Raman spectroscopy of the g-CuNWs. (c) XRD spectra of the CuNWs and g-CuNWs. (d) Contact angle measurements of the CuNWs and g-CuNWs.

In Figure 1c,f, we utilize the free-standing vertically aligned Cu nanowire (CuNW) array (grown on a thin Cu film) as a scaffold for graphene synthesis, where the CuNWs are fabricated by Cu electroplating in a porous anodic aluminum oxide template with subsequent wet etching and supercritical drying. The high-aspect-ratio CuNWs are mechanically compliant with a typical diameter and length of ∼150 nm and ∼20 μm, respectively (see Methods for detailed fabrication). The selection of nanowire dimensions is a trade-off between thermal and mechanical performances. While a shorter length of nanowires is chosen to meet the usual bond line thickness, slenderness (smaller diameter) is necessary to ensure high mechanical compliance. At the same time, the nanowires cannot be too thin to enter the nanoscale size-dependent thermal transport regime, in which the thermal conductivity of individual nanowires is significantly reduced compared with the bulk thermal conductivity. Through our customized plasma-enhanced chemical vapor deposition (PECVD) system^35^ (see Methods for detailed synthesis parameters), dense single- to few-layer graphene flakes are conformally coated on the entire surface of the CuNWs (Figure 1d,g), as illustrated by the energy-dispersive spectroscopy (EDS) mapping (Figure 2a), while relatively more graphene is found at the tips of the CuNWs because of the higher local reactive gas concentration. In Figure 2b, the representative peaks (i.e., D, G, and 2D peaks) in the Raman spectrum (see Methods for detailed description) further confirm the existence of graphene. The dense and conformal graphene flakes can not only enhance the thermal conductivity of CuNWs but also effectively prevent them from oxidation in air. As revealed by the X-ray diffraction (XRD) spectra in Figure 2c, compared with the as-fabricated CuNWs that can be quickly oxidized by air, there is no oxide observed in the g-CuNWs even after 6 months (see Methods for characterization details). More importantly, after the graphene coating, the wetting behavior of CuNWs can be dramatically transformed from superhydrophilic with a nearly 0° contact angle to hydrophobic with a contact angle of 135.7°, as shown by the contact angle measurements in Figure 2d for bare CuNWs and g-CuNWs, respectively. Such hydrophobicity is crucial for allowing the electrochemical deposition of a thin and continuous Cu layer around just the nanowire tips to protect and strongly bond to the g-CuNWs, thus forming a 3D graphene-nanowire “sandwich” structure (Figure 1e,h). To introduce solderability, thin Sn layers (3–5 μm thick) are directly deposited on the top and the bottom Cu layers, respectively, without affecting the g-CuNWs. The final structure is free-standing and flexible like paper with a total thickness of <40 μm.

**Figure 3:**
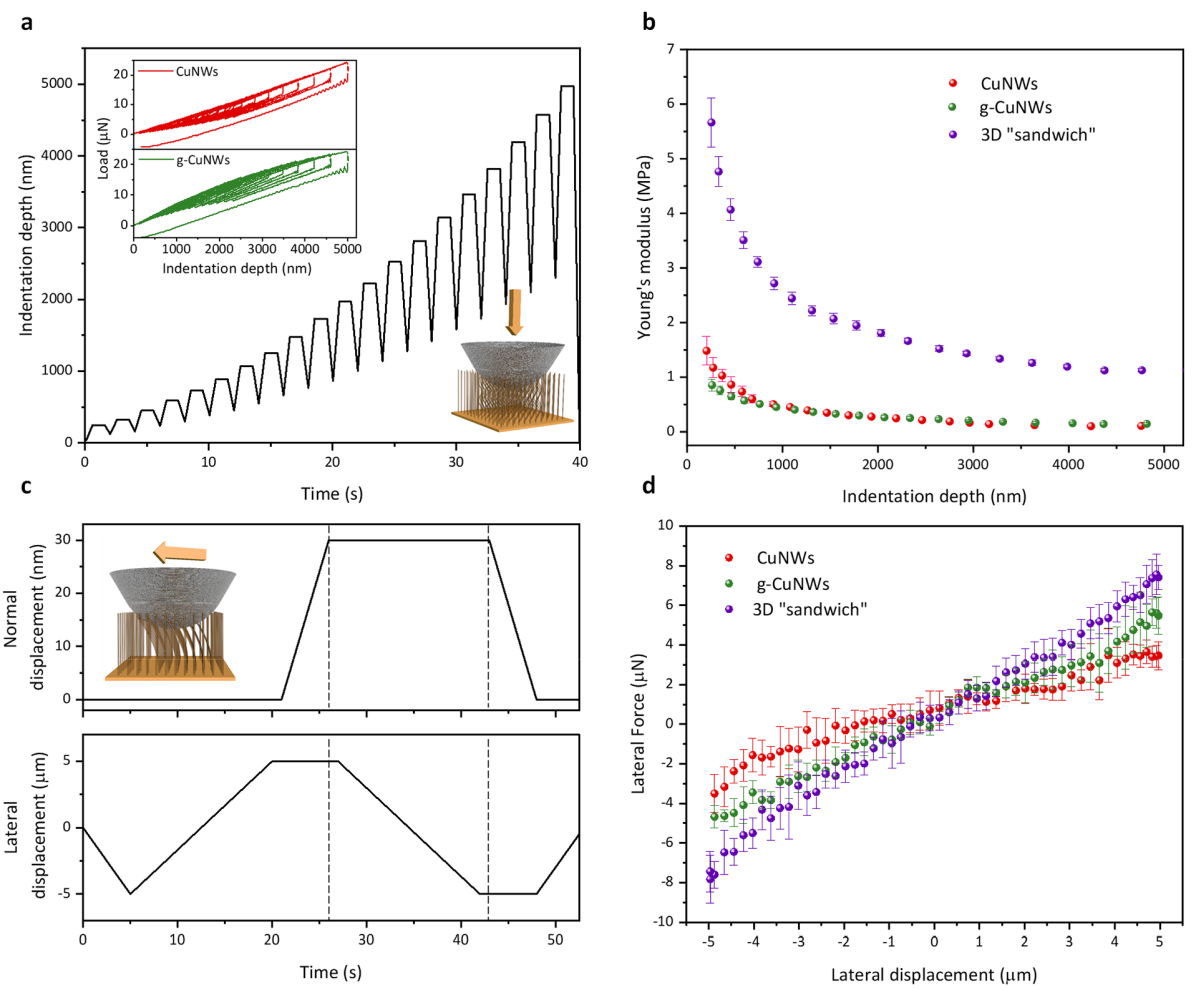
Mechanical characterization of CuNW array, g-CuNW array, and 3D “sandwich.” (a) Quasi-static load–partial unload tests (20 cycles) with controlled normal displacement for Young’s modulus measurements. Inset on the upper left: representative load–displacement curves. Inset on the lower right: schematic of the indentation test. (b) Measured Young’s moduli calculated from the Oliver–Pharr model. (c) Load functions of a scratch test. Within the dashed line framed region, a normal displacement of 30 nm is exerted by the probe tip after the initial touch to make full contact, and then the probe moves laterally from +5 μm to −5 μm. Inset: schematic of the scratch test. (d) Measured lateral force versus lateral displacement. The error bars in (b) and (d) are the standard deviation from multiple measurements.

In order to quantify the compliance of the 3D graphene-nanowire “sandwich” structure, we characterize its mechanical properties using a nanoindentor (Hysitron TI900 Triboindenter) and compare it with the cases of bare CuNWs and g-CuNWs (see Supplementary Note S1 for detailed mechanical characterization). For the Young’s modulus measurements, the load–partial unload method with controlled normal displacement is performed for 20 cycles (Figure 3a). This method can accurately evaluate the Young’s modulus of materials because it minimizes the indentation size effect.^36^ Through the application of the Oliver–Pharr model^37^ on each cycle, the nominal Young’s moduli of bare CuNWs, g-CuNWs, and the 3D “sandwich” structure can be calculated as a function of indentation depth, as illustrated in Figure 3b. For all three cases, the calculated Young’s moduli first decrease with increased indentation depth and then reach a plateau at the depth of ∼4 μm, which indicates that the indentation size effect can be neglected for depths >4 μm. The Young’s moduli determined on the plateau are 0.123 ± 0.006 MPa for bare CuNWs, 0.145 ± 0.017 MPa for g-CuNWs, and 1.35 ± 0.026 MPa for the 3D “sandwich” structure, which is 2 orders of magnitude lower than previous work.^29^ To explain such significant drop-off, while the bulk behavior of the nanowire-based structures is determined from the indentation using the Oliver–Pharr model, the deformation of each nanowire can be understood by classical beam bending predicted by the Euler–Bernoulli beam theory with pin-fixed boundary conditions.^38−40^ The reason why each nanowire can be treated as an individual slender beam is attributed to the vertically well-aligned nanowires with almost no neighboring interactions. Because of their higher aspect ratio and much lower density, the measured low stiffness is a reasonable estimation (see Supplementary Note S1 for detailed analysis and supporting evidence). For the shear modulus measurements, we conduct standard scratch tests in which, after the probe tip contacts the specimen surfaces, a normal displacement of 30 nm is exerted on the samples to ensure full contact while the probe moves laterally (Figure 3c). By measuring the lateral force as a function of lateral displacement (Figure 3d), the shear modulus can be estimated using *Fl/*(*A*Δ*x*), where *F* is the lateral force, Δ*x* is the lateral displacement, *l* is the thickness of the sample, and *A* is the shearing area that is determined by contact probe area. The measured shear moduli of bare CuNWs, g-CuNWs, and the 3D “sandwich” structure are 0.447 ± 0.275, 0.738 ± 0.236, and 1.044 ± 0.289 MPa, respectively. Both the measured Young’s modulus (1.35 MPa) and shear modulus (1.044 MPa) are about 4 orders of magnitude smaller than those of solder alloys (10–20 GPa) and manifest that the 3D graphene-nanowire “sandwich” structure has a low stiffness like foams and polymers.

**Figure 4:**
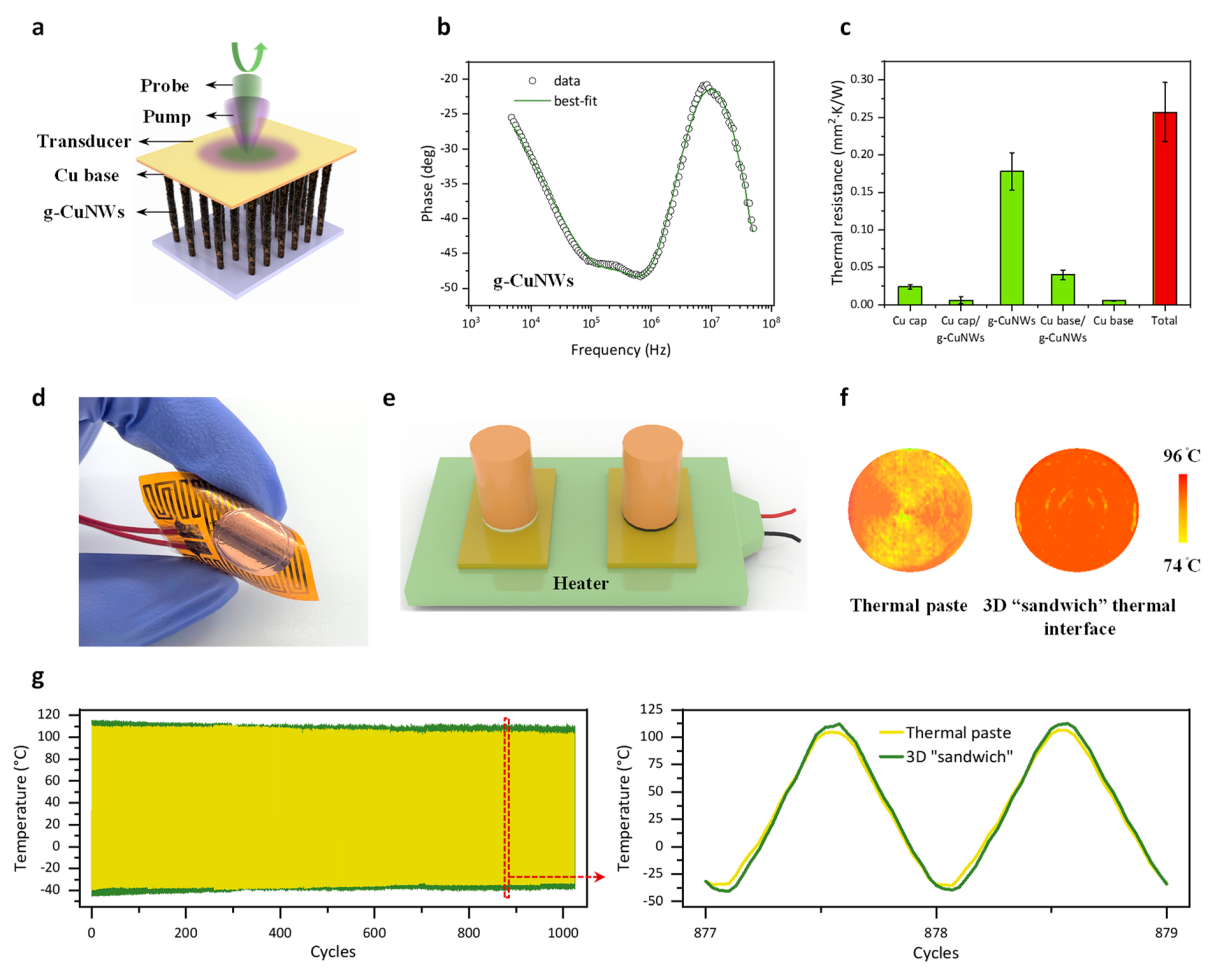
Thermal and reliability characterization of the 3D graphene-nanowire “sandwich” TIM. (a) Configuration of the frequency-domain thermoreflectance (FDTR) measurement. (b) Experimental data and best least-squares fitting to the heat transfer model for extracting the thermal conductivities of g-CuNWs and Cu base/g-CuNWs interfacial conductance (see Supplementary Note S2 for detailed measurements). (c) Thermal resistance histogram of the “sandwich” structure. (d) The 3D “sandwich” soldered to a flexible heater. (e,f) Thermal infrared mapping for the comparison between a commercial thermal paste and the 3D “sandwich” TIM with the same dimension. For the same heater temperature, the sample jointed by the 3D “sandwich” demonstrates a higher temperature close to the heater and, thus, a lower overall thermal resistance compared with the one jointed by the thermal paste. (g) Thermal cycling test between −55 °C to 125 °C. The right panel shows the representative temperature cycle profile. The left panel illustrates >1000 temperature cycles with high consistency, thereby indicating the excellent long-term stability of the 3D graphene-nanowire “sandwich” thermal interface.

We implement the frequency-domain thermoreflectance (FDTR) method to measure the thermal transport properties of the fabricated 3D graphene-nanowire “sandwich” structure. As shown in Figure 4a, the FDTR method is a pump–probe optical technique for measuring thermal properties on the basis of different frequency-domain thermal responses of materials. The modulated pump beam excites the sample, whereas the probe beam measures the changes in the temperature-dependent reflectance. The phase lag induced between a reference input from the pump laser and the reflected probe laser is measured as a function of the modulation frequency. The thermal properties, including thermal conductivity, anisotropy ratio, and thermal interface resistance of the sample, can be extracted by fitting the obtained data using a 2D heat conduction model for multilayer thin films.^41^ For an array of bare CuNWs, the typical data (phase lag as a function of modulation frequency) and the best fit from the heat transfer model give the thermal conductivity of CuNWs to be 65 ± 6 W/m·K, which is consistent with the estimation based on the ∼20% CuNW filling ratio (detailed procedure to validate the measurement has been elaborated in Supplementary Note S2). After the graphene deposition on the CuNW sample (the fitting is demonstrated as Figure 4b), its thermal conductivity is dramatically enhanced to be 97 ± 14 W/m·K (Sample #1), which represents an ∼50% improvement. We also measure the thermal conductivities of the other two g-CuNW samples to be 112 ± 16 W/m·K (Sample #2) and 128 ± 19 W/m·K (Sample #3). The corresponding overall thermal resistances of the three graphene-nanowire “sandwich” samples are ∼0.27 mm^2^·K/W (Sample #1), ∼0.26 mm^2^·K/W (Sample #2), and ∼0.24 mm^2^·K/W (Sample #3) (see Supplementary Note S2 for the detailed characterization procedure of interfacial resistances and Table S2 for data summary). The ultralow thermal resistance (∼0.24 mm^2^·K/W) is about 1 order of magnitude smaller than that of conventional solders (see Supplementary Notes S3 and S4 for uncertainty and sensitivity analysis). It is worth noting that for the three samples, the average thermal interface resistance (∼0.006 mm^2^·K/W) between the g-CuNWs and their electroplated Cu cap layers is about 1 order of magnitude smaller than that between the as-grown CuNWs and their original Cu base layers (∼0.04 mm^2^·K/W), which demonstrates the excellent bonding of the g-CuNWs with the electroplated Cu layer.

To further demonstrate the superior performance of the 3D graphene-nanowire “sandwich” thermal interface, in Figure 4d, we solder the sample to a flexible heater, bond it to a Cu block as the heat spreader, and compare its cooling results with a thermal paste that has the same dimensions (see Supplementary Note S5 for detailed setup description). This controlled group of two assemblies is mounted on the same heater with an area of 35 mm × 20 mm and 50 W applied. Immediately after the heater is switched on, the top surface temperature of the assemblies is measured by an infrared camera. From the infrared temperature mapping (Figure 4e,f) result, we can clearly see that the 3D “sandwich” TIM renders a smaller top temperature difference from the heater and, thus, lower resistance compared with the thermal paste. Because interfaces are subject to cyclic heating/cooling that emulates on/off working conditions of electronics, the thermal stress generated at interfaces can induce cumulative fatigue and even cause device degradation and failure. The long-term reliability of a thermal interface is, therefore, vital for the performance of electronics. To evaluate the reliability of the 3D “sandwich” thermal interface, we solder the sample to bond Si and Cu substrates and conduct the temperature cycling test in a wide temperature range from −55 °C to 125 °C, in which one thermocouple is attached on the top to monitor the temperature (see Supplementary Note S6 for detailed setup description). As a control experiment, a similar assembly by a commercial thermal paste experiencing the same cycle is measured, as well. One thermal cycle includes a 2 min ramping up from −55 °C to 125 °C, a 30 s dwelling, a 2 min ramping down from 125 °C to −55 °C, and another 30 s dwelling, as shown in Figure 4g. The measured temperatures exhibit excellent stability after >1000 cycles (Figure 4g), which, according to the industry standard,^42,43^ is an indication of long-term reliability, thus demonstrating the long-term reliability of the 3D graphene-nanowire “sandwich” thermal interface. Moreover, in comparison with the assembly integrated with the thermal paste, the “sandwich” assembly possesses a higher temperature range, thereby indicating the higher through-plane thermal transport.

## Conclusion

In summary, we demonstrate a free-standing, paperlike, and flexible 3D graphene-nanowire “sandwich” TIM that enables excellent solderability and has ultrahigh mechanical compliance, like polymers and foams, with an ultralow thermal resistance about 1 order of magnitude smaller than that of traditional solders. From temperature cycling tests in a wide temperature range, the 3D “sandwich” shows good long-term reliability. Since most flexible electronics (e.g., on Kapton tapes) and microelectronics are solderable, the 3D “sandwich” TIM demonstrated in this work can be applied to a broad range of flexible and curved surfaces in electronics for advanced thermal management, energy conversion, and energy harvesting technologies.

## Materials and Methods

### Fabrication of the 3D Graphene-Nanowire “Sandwich” TIM

A thin layer of copper (∼2 μm), labeled as “Cu base,” was sputtered on the anodic aluminum oxide (AAO) template (140 nm pore size, 450 nm interpore distance, 50 μm in thickness) to form a seed layer for the subsequent copper nanowire electroplating. To have copper electrodeposited only from the open hole side, a sample holder that blocks the Cu base was exploited. In the copper electrolyte bath (Sigma-Aldrich), a square wave current was applied whereby the “on” time determines the copper nanowire length (∼20 μm). The AAO template was then dissolved in KOH solution to release the nanowires. Concurrently, critical-point drying (CPD) was conducted to remove the solution, by such manner that the nanowire aggregation caused by liquid surface tension can be avoided. Subsequently, the out-of-plane graphene was synthesized on the copper nanowire arrays by PECVD method. A homemade fixture was designed for holding the CuNWs/Cu base in the reaction gas flow composed of methane and argon (CH_4_/Ar = 1:19, 50 sccm). Following the PECVD synthesis, the copper electroplating was conducted again, in which the copper was only deposited on the tip of the g-CuNWs to form an ∼8 μm thick continuous layer, labeled as “Cu cap.” The “sandwich” structure was enabled to spread and bond with other substrates by electroplating thin tin layers on the top and the bottom of the material. Via trial and error, a tin layer of 3–5 μm was determined sufficient to combine two relative rough surfaces. During assembly, it was sandwiched between two substrates with a mild pressure applied and heated to the melting point of tin (∼240 °C) for few seconds. The melted tin was able to flow and conform to the roughness of the mating surfaces and join them together after cooling down. As opposed to the conventional ≥20 μm soldering bond line thickness, the reason why such a thin tin layer can be used here is attributed to the flexible nanowires that can readily conform to the major surface asperities, while the liquid tin only needs to fill the remaining tiny air voids.

### Optimization of the Low-Temperature PECVD Process

In the process of graphene synthesis, it is worth noting that the CuNWs possess a lower melting point than bulk copper because of the size effect. In this work, we adopted low-temperature PECVD,^44,45^ which enables the graphene synthesis at a temperature as low as 500 °C. In addition to the temperature, other synthesis conditions were optimized for this application, as well. As shown from the EDS (Quanta 600) mapping (Figure 2a), the CuNWs were entirely coated with carbon. However, there was a decreasing carbon density distribution from the nanowire tip to the bottom. Such nonuniformity is because of the extremely high aspect ratio and relatively high filling ratio of the nanowires. At the beginning of the synthesis, the CH_4_ molecules are able to precipitate and form conformal deposition, whereas, as the process carries on, the CuNW–graphene core–shell structure becomes thicker, which contributes to even narrower nanogaps between the neighboring nanostructures. Consequently, it poses challenges for CH_4_ molecules to further sink. To increase the gas diffusion, a lower pressure is preferred because the lower the pressure is, the longer the mean free path of the reactant molecules will be. However, a relatively high pressure is in favor of the deposition rate. As a compromise, 0.9 Torr for the total pressure, i.e., 45 mTorr for the partial pressure of methane, was employed in this work. Furthermore, despite the benefit of RF plasma on the deposition rate, a high plasma power results in more molecule collisions that will amplify the nonuniformity of the graphene coating. Here, the 60 W RF power for a 4 h synthesis was used.

### Raman Spectroscopy of g-CuNWs

Details with regards to the structure and quality of the out-of-plane graphene flakes were examined by Raman spectroscopy (NT-MDT NTEGRA). The representative peaks in the Raman spectrum (Figure 2b), i.e., D, G, and 2D peaks, confirm the existence of graphene. The typical G band around 1580 cm^–1^ is generated by the E_2g_ phonon at the center of the Brillouin zone, while the 2D band at ∼2680 cm^–1^ representing the double-resonant Raman scattering can be explained by the appearance of juxtaposed single- to few-layer graphene flakes in the form of high-density graphene flakes. The strong D band at ∼1340 cm^–1^ that indicates structural disorders and defects is also observed. While the D band is generated from the one-phonon defect-assisted process, the D + D′ band corresponds to a two-phonon defect-assisted process.^46^ For the out-of-plane graphene flakes, the presence of edges causes translational symmetry breaking, which results in the emergence of the D′ peak as a shoulder to the G peak.

### X-Ray Diffraction Spectroscopy of CuNWs Embedded in the AAO, Bare CuNWs, and g-CuNWs

The X-ray diffraction (XRD) spectroscopy (Empyrean) was used to investigate the composition of both CuNWs and g-CuNWs. A benchmark XRD spectrum of CuNWs embedded in the AAO was also performed (Figure S11), from which only Cu was observed. After the removal of the AAO, however, CuNWs were quickly oxidized to form copper oxide and cuprous oxide, as illustrated by the XRD peaks at 36.5°, 42°, and 61.5° (Figure 2c). In contrast, there are no XRD peaks of the oxides observed for the g-CuNWs, even after more than six months in air. This is because the presence of hydrogen gas during the PECVD process completely removes the oxide layer on the CuNWs’ surfaces. More importantly, the dense out-of-plane graphene flakes effectively prevent the nanowires from oxidation and contribute to the excellent stability of the material.

### Specific Heat Capacity Characterization of CuNWs and g-CuNWs

To extract thermal properties from FDTR measurements, it is important to know the specific heat of the material, including CuNWs and g-CuNWs. This property was quantified by exploiting the TA Instruments Discovery DSC250 Differential Scanning Calorimeter (DSC). Prior to the measurement, the CuNWs on Cu base and the g-CuNWs on Cu base samples were pressed and sealed in separate aluminum pans. The measurement scans were performed in the order of empty baseline, sapphire disk, and unknown samples. All of them experienced the same procedure: room temperature to 200 °C with a ramp rate of 20 °C/min and isothermal for 10 min. Given the known masses of sapphire and the sample and the well-known sapphire heat capacity, the heat capacity of the unknown materials could be determined by the heat flow difference with the sapphire. As a result, the specific heat values of the CuNWs on the Cu base and g-CuNWs on the Cu base were measured to be 0.31 and 0.3 J/g·K, respectively, at room temperature. After excluding the contribution from the Cu base layer, the specific heat values of the CuNWs and g-CuNWs were estimated to be 0.287 and 0.273 J/g·K. The density was evaluated on the basis of the mass and volume to determine the volumetric specific heat of the CuNWs and g-CuNWs.

## Supporting Information Available

Supplementary Notes S1–S6: Detailed characterization of mechanical properties, brief introduction of the FDTR method and FDTR measurement on different materials, FDTR measurement error analysis, FDTR measurement sensitivity analysis, infrared thermal mapping, and temperature cycling test. Figures S1–S11: Image of the nanoindenter probe; load–displacement curves; schematic illustration of frequency-domain thermoreflectance (FDTR) setup; FDTR data plots with best fits of silicon, quartz, and copper; measurement configuration of the 3D “sandwich” structure with experimental data and best fit; SEM top-view images of original Cu base layer and Cu base layer after ion milling; FDTR fitting sensitivity analysis; schematic of experimental setup for infrared thermal mapping; time-dependent COMSOL simulation of thermal mapping; schematic of experimental setup for temperature cycling test; and X-ray diffraction patterns. Tables S1 and S2: Summary of Young’s modulus and shear modulus data with standard deviation from multiple measurements and summary of fitted results on the basis of CuNWs, g-CuNWs, and 3D “sandwich” thermal measurements (PDF)
