# Supplementary material for: 3D Graphene-Nanowire “Sandwich” Thermal Interface with Ultralow Resistance and Stiffness
Source: ACS Nano. 2023 Jan 17;17(3):2602–10. doi: 10.1021/acsnano.2c10525 (PMC10041630; doi:10.1021/acsnano.2c10525)
Supplement: Supplementary file 1 — nn2c10525_si_001.pdf [file nn2c10525_si_001.pdf]

Supplementary Materials for

3D Graphene-Nanowire "sandwich" Thermal Interface with Ultra-Low Resistance and Stiffness

*Lin Jing[1†], Rui Cheng[1†], Raghav Garg[2], Wei Gong[1], Inkyu Lee[2], Aaron Schmit[3], Tzahi Cohen-Karni[2], Xu Zhang[4], Sheng Shen[1]**

[1] Department of Mechanical Engineering, Carnegie Mellon University; Pittsburgh, PA 15213 USA

[2] Department of Materials Science and Engineering, Carnegie Mellon University; Pittsburgh, PA 15213 USA

[3] Department of Mechanical Engineering, Massachusetts Institute of Technology; Cambridge, MA 02139 USA

[4] Department of Electrical and Computer Engineering, Carnegie Mellon University; Pittsburgh, PA 15213 USA

† These authors contributed equally to this work.

* Corresponding author. E-mail: sshen1@cmu.edu

This PDF file includes:

Supplementary Note S1 to S6
Figure S1 to S11
Table S1 to S2
References [1-8]

Other Supplementary Materials for this manuscript include the following:

Supplementary Note S1: Detailed characterization of mechanical properties

In this work, both Young's modulus and shear modulus were characterized using a nanoindenter (Hysitron TI900 Triboindenter) equipped with a 100 µm (radius), ~ 90° conical probe (**Figure S1**), by which tests were made on the CuNWs, g-CuNWs and the 3D "sandwich".

In the Young's modulus measurements, 20 cycles of load-partial unload with controlled normal displacement starting from 0.25 µm and ending at 5 µm were performed. The obtained load-displacement curve for CuNWs, g-CuNWs, and the "sandwich" structure are demonstrated in **Figure S2**. By adopting the Oliver-Pharr model, the nominal Young's modulus can be calculated as a function of indentation depth, as illustrated in **Figure 3(b)**. It is clear that the nominal compressive modulus under all the circumstances begins with higher values and reaches a plateau at the depth of ~ 4 µm. Accordingly, it is confirmed that there is negligible indentation size effect at the depths > 4 µm,[1,2] where the real Young's modulus is determined as shown in **Table S1**. What is happening during the indentation is that upon the loading, the vertically aligned nanowires will bend to accommodate the stress. While the bulk behavior of the nanowire-based structures is determined from the load-depth curve using the Oliver-Pharr model, the deformation of each nanowire can be understood by classical beam bending predicted by Euler-Bernoulli beam theory with pin-fixed boundary conditions (Eq. (1)). The reason why each individual nanowire can be treated as classical vertical slender column is attributed to the vertically well-aligned nanowires with hardly any neighboring interactions.

$$\delta_{max} = \frac{PL^3}{3EI}, where\ I = \frac{\pi d^4}{64}. \quad (1)$$

By substituting the nanowire dimensions in[3], ~ 12 µm long and ~ 210 nm, and those in this work, ~ 20 µm long and ~ 150 nm, respectively, to Eq. (1), we can know the much slender nanowire in

this paper will undergo roughly 20 times higher deflection. Namely, only from the single nanowire perspective, the much thinner nanowire in this work will result in 20 times lower stiffness. More importantly, even it is claimed as 20% volume ratio in[3], the nanowires are much denser than its nominal value. From the observation under SEM, the spacing between two adjacent nanowires in[3] is ~ 350 nm, while it is ~ 450 nm in this work. Hence, there are not only more interactions among neighboring nanowires, but it's also in high density, which will both contribute to greater bulk mechanical resistance for the earlier work. All these factors combined will explain the significant modulus reduce in comparison with our previous work.

In comparison with CuNWs, g-CuNWs exhibits lower mechanical resistance at the early stage of indentation but behaves similarly on the plateau. This stems from the fact that, for g-CuNWs, the initial contact of the probe is made with the porous and soft graphene flakes covered at the tip of CuNWs, thus demonstrating higher compliance at the beginning. Regardless, ultralow Young's moduli (0.123 MPa and 0.145 MPa) are obtained for CuNWs and g-CuNWs, respectively. It hence proves that the coating of these out-of-plane graphene flakes can barely degrade the compressive mechanical performance of CuNWs. For the 3D "sandwich", the measured Young's modulus (1.350 MPa) is about one order of magnitude larger under the same measurement condition, compared with CuNWs and g-CuNWs.

As shown in **Figure 3(c)**, scratch tests were conducted to examine the shear modulus. Immediately after the probe tip contacts the sample surface, a normal displacement of 30 nm is exerted to ensure full contact with the nanowires. Concurrently, the probe moves laterally from +5 μm to -5 μm. As a result, the lateral force versus lateral displacement (**Figure 3(d)**) during the period framed by dashed lines in Figure 3(c) is utilized to estimate the shear modulus by:

$$G = \frac{Fl}{A\Delta x}, \quad (2)$$

where F is the lateral force, Δx is the lateral displacement, l is the total thickness of the sample, and A is the shearing area determined by contact probe area. For a conical probe (Figure S1), the shearing area A is expressed as:

$$A = -\pi h_c^2 + 2\pi R h_c, \quad (3)$$

where R is the radius of the probe (100.24 μm), and h_c is the normal displacement (30 nm). A is thus calculated to be 18.892 μm^2. It is worth noting that in Figure 3(d) the lateral force linearly relates to the lateral displacement for all the cases. Due to the nonuniformity of the nanowire length and the relatively rough Cu cap layer, the scratch tests show relatively large uncertainties. Nevertheless, among all the cases, CuNWs shows the lowest shear modulus of 0.447 MPa, while the coating of graphene flakes slightly densifies the nanowires and thus leads to 0.738 MPa for g-CuNWs. For the "sandwich" structure, the relatively thick Cu cap layer (~ 6-8 μm) gives rise to approximately 41% higher shear modulus as compared with g-CuNWs. All the measured shear modulus data are summarized in Table S1.

Supplementary Note S2: Brief introduction of the FDTR method and FDTR measurement on different materials

A frequency-domain thermoreflectance (FDTR) system from Fourier Scientific was used to investigate the thermal conductivity and boundary conductance of each layer of the "sandwich" structure, by which the total resistance can be derived via taking each part into account. The FDTR method is a pump-probe optical technique for measuring thermal properties grounded on the frequency-domain thermal response of materials (**Figure S3**). The pump beam (405 nm in wavelength) with modulated frequencies excites the sample and the probe beam (532 nm in

wavelength) measures the changes in the temperature-dependent reflectivity. The phase lag induced between a reference input from the pump laser and the reflected probe laser is collected as a function of the modulation frequency. When analyzing the data, it requires the fitting between the obtained data and the heat transfer model, which, in this case, is 2D heat conduction for multilayer thin films with a cylindrical heat source. The unknown thermal properties are treated as free parameters and adjusted to minimize the discrepancy to achieve the best fit (detailed descriptions and math deviations can be found in Refs. (3, 4)). During the measurement, a thin metal film is deposited on the top of the sample as an energy absorber for the pump beam and the temperature transducer for the probe beam. If the probe laser of a specific wavelength is employed, the metal film material must be carefully chosen so that it possesses a large thermoreflectance coefficient at this wavelength. Gold is selected as the transducer material in this work as the 532 nm probe laser is used.

Prior to the investigation on the samples, the accuracy of the system was firstly verified by measuring well-known materials, such as silicon and quartz (**Figure S4 (a-b)**). Their resulting thermal conductivities are 147 W/m·K and 3.1 W/m·K, respectively. After that, we measured the thermal conductivity of the Cu base layer as a verification for subsequent measurements (**Figure S4 (c)**). In this step, the Cu layer following the same preparation procedure as that in CuNWs or g-CuNWs was sputtered on a Si substrate. In the tri-layer configuration (Au/Cu/Si), the boundary conductances of Au/Cu and Cu/Si, the Cu thermal conductivity, and the Cu layer thickness were determined to be 1.5e8 ± 1.2e6 $W/m^2 \cdot K$, 9.4e7 ± 1.2e7 $W/m^2 \cdot K$, 322 ± 52 W/m·K, and 1.6e-6 ± 1.1e-7 m (which is consistent with the measurement from SEM), respectively, by fitting the data in Figure S4 (c).

The CuNWs and g-CuNWs thermal conductivities and their corresponding boundary conductances with the Cu base layer were characterized by the Au/Cu base/NWs multilayer/insulation layer configuration as shown in **Figure 4(a),** and the results are presented in **Table S2**. With an insulation layer underneath, the nanowire layer thickness was fitted as well. As a result, it was determined to be ~ 16 μm, which agrees well with the observation from SEM. The resulting copper base thermal conductivities are also consistent with that was solely obtained (322 W/m·K) within the uncertainty range. In order to examine the boundary conductance *G* between the Cu cap layer and g-CuNWs, the measurement was performed on the Cu cap side instead, as shown in **Figure S5**. In this four-layer structure, as all other parameters were acquired from the previous characterizations, the Cu cap thermal conductivity and Cu cap to g-CuNWs interfacial conductance were fitted (Table S2) in addition to the g-CuNWs thermal conductivity, the resulting value of which is in accordance with that was obtained above by Figure 4(a). Via accounting for thermal conductivities of each layer and all interfacial conductances, the total resistance of the 3D "sandwich" is obtained.

Based on the configurations in Figure 4(a) and **Figure S5(a)**, we conducted the FDTR measurements on three separate "sandwich" samples, and the results are summarized in Table S2**.** The error bars here come from the non-linear least-squares fitting process.

Supplementary Note S3: FDTR measurement error analysis

In the thermal characterization, there are several factors that need to be considered to achieve accurate results. One is from the FDTR setup, including the presence of odd harmonic components of the fundamental frequency in the measured signal and errors from the measurement of the focused size of the pump and probe beams. These problems have been addressed and corresponding solutions to improve the measurement accuracy can be found in reference. In this

work, another factor contributing to uncertainties is from the sample. Accordingly, multiple measurements at different spots as well as multiple data collection on the same spot were implemented and averaged in order to reduce the regression uncertainty. Furthermore, to improve the reflective signal-to-noise ratio, ion milling (Commonwealth Scientific) was performed on the Cu layer to obtain a flat and smooth surface before the gold transducer was deposited, as shown in **Figure S6.** The accurate characterization of the gold transducer layer thickness was done by using a reference Si substrate and placing it next to the samples in the deposition chamber, which is also in favor of the accuracy. Combining the uncertainty from the goodness-of-fit, input parameter uncertainties including the measured laser beam size, specific heat capacity, and copper layer thickness, and the measurement uncertainty on different samples,[6–8] the overall uncertainty of the resulting thermal conductivity *k* of g-CuNWs, boundary conductance *G* of Cu base to g-CuNWs, and boundary conductance *G* of Cu cap to g-CuNWs are ~25%, ~28%, and ~85%, respectively.

Supplementary Note S4: FDTR Measurement sensitivity analysis

The sensitivity of obtained phase signal ϕ to an interested parameter *x* was evaluated as follows[5]:

$$S_x = \frac{d\phi}{dlnx}. \quad (4)$$

The properties that were simultaneously extracted from one data set involve the Cu layer (Cu base or cap) thermal conductivity k, thermal boundary conductance *G* between the Cu layer and nanowires, *k* of nanowires, and nanowire array thickness *t*. Therefore, except for the nanowire array thickness whose accuracy was verified by the SEM, these parameters were considered for sensitivity evaluations under three measurement configurations (**Figure S7**).

The thermal transport model applied here is the two-dimensional heat conduction along the cross-plane and radial directions with a Gaussian heat source. However, at high frequencies, the thermal

penetration depth $\sqrt{2\alpha/\omega}$ in the material (where α is the thermal diffusivity) will be much smaller as compared to the pump laser spot size, which is 16.35 μm here. Thus, it will approach one-dimensional heat diffusion along the cross-plane direction and only cross-plane thermal conductivity plays a role. For this reason, the anisotropy of the nanowire is insensitive in the majority of the frequency range and therefore not considered here. From Figure S7, except for the boundary conductance between the Cu cap and g-CuNWs in the configuration (c), the sensitivities of these three parameters maintain significant and different in the frequency range for (a), (b), and (c) and confirm that their fittings from one set of data are feasible and reasonable. With regards to G between the Cu cap and g-CuNWs, it is the relatively thick Cu layer that induces its low sensitivity. But it still varies and differs from the other two within the frequency range and therefore can be simultaneously extracted.

Supplementary Note S5: Infrared thermal mapping

As shown in **Figure S8**, the 3D "sandwich" and a commercially available thermal paste (Vetroo INC) with the same dimension (8 mm in diameter, 40 μm in thickness) were used to respectively bond a Si substrate (15 mm in length, 10 mm in width, 0.5 mm in height) with a Cu block (8 mm in diameter, 15 mm in height) as the heat sink. With these two assemblies mounted on the same heater at the same initial room temperature, immediately after the heater was switched on, the temperatures of the top surfaces of the assemblies were measured by an infrared camera (QFI InfraScope™). Compared to the thermal paste, the top surface temperature of the assembly by the "sandwich" material increased more rapidly to reach a value of ~ 7 °C higher than that by the thermal paste (**Figure 4(e-f)**). This can be understood via fin problem simplification. The general solution to the fin differential equation can be expressed as:

$$\theta = C_1 e^{mx} + C_2 e^{-mx}, m = \sqrt{\frac{hp}{kA_c}} \quad (5)$$

Where m^{-1} has a unit of m, which can approximately serve as the temperature decay length. When k is higher, the temperature decay from the base will be mild, *i.e.,* the tip temperature will be higher.

Accordingly, the finite element simulation (COMSOL) was conducted to get the insight more straightforwardly. In the transient simulation, all boundary conditions are set according to the real situation. For the two cases, everything is identical except in one case, the interface material is made of standard thermal grease with k of 3 W/m·K, while in other case, it is the “sandwich” of 100 W/ m·K. Other properties about the “sandwich” including heat capacity and density are obtained via DSC measurement and volume ratio calculation, as elaborated in the Materials and Methods section. As shown in Figure. S9, it can be seen that in the first 15 s after the heater is switched on, the top surface temperature of the assembly by the “sandwich” is ~ 4 °C higher than that by thermal paste, while the difference is ~ 7 °C in the thermal mapping. Such deviation can be explained from the artifacts and experiment errors. Even there is approximate 100 times contrast in thermal conductivity, the induced tip temperature difference is little because the interface is too thin to account for high difference. However, the resulted difference will be significant when the power is higher, *e.g.,* 100 W, as shown in Figure. S9(b). Therefore, the advantage of the high conductivity “sandwich” material can be maximized at high power.

Supplementary Note S6: Temperature cycling test

As shown in **Figure S10**, the temperature cycling was conducted to examine the reliability of the 3D “sandwich” and make comparison to thermal paste. In the test, silicon (10 mm in length, 10 mm in width, 0.5 mm in height) and copper substrates (10 mm in length, 10 mm in width, 1 mm

in height) were bonded by the “sandwich” (10 mm in length, 10 mm in width, 40 μm in height), as well as the same sized thermal paste. The two assemblies were then mounted on the Linkam stage (HFS600E) equipped with a liquid nitrogen pump (LNP96) to control the temperature precisely and rapidly. The profile of the cycle applied to the stage is as follows: 2 min from -55 °C to 125 °C and dwelling for 30 s, then 2 min ramping down to -55 °C and keeping the temperature for another 30 s. The live temperature on the top of two assemblies were monitored by thermocouples that were fixed on the top surfaces. As presented in **Figure 4(g)**, the upper surface temperature exhibits excellent stability even after >1000 cycles, which accordingly, indicates the long-term reliability of the 3D “sandwich” thermal interface.

Figure S1.

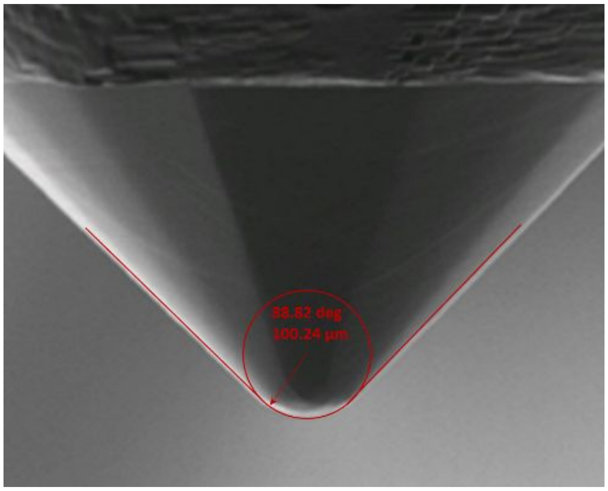

Figure S1. Image of the nanoindenter probe.

Figure S2.

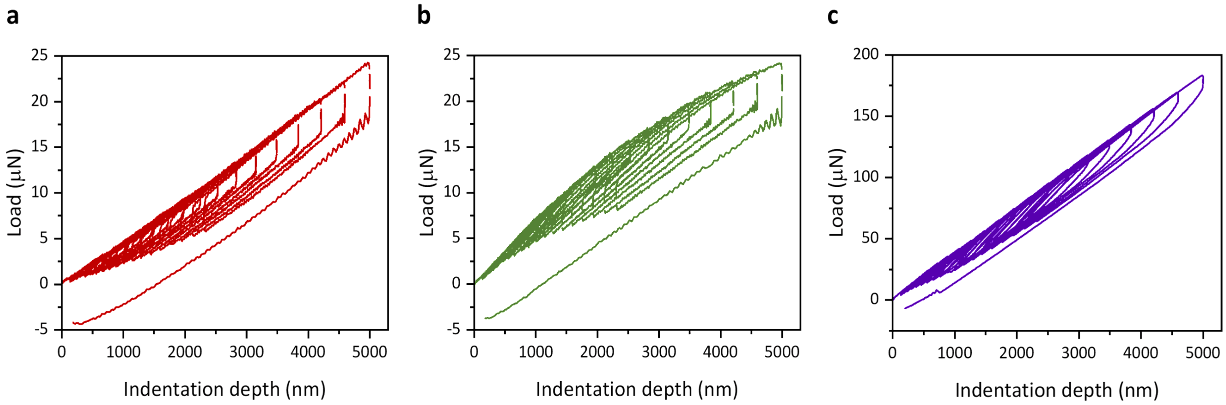

Figure S2. Load-displacement curves of a, CuNWs on the Cu base, **b**, g-CuNWs on the Cu base, **c**, 3D "sandwich" structure.

Figure S3.

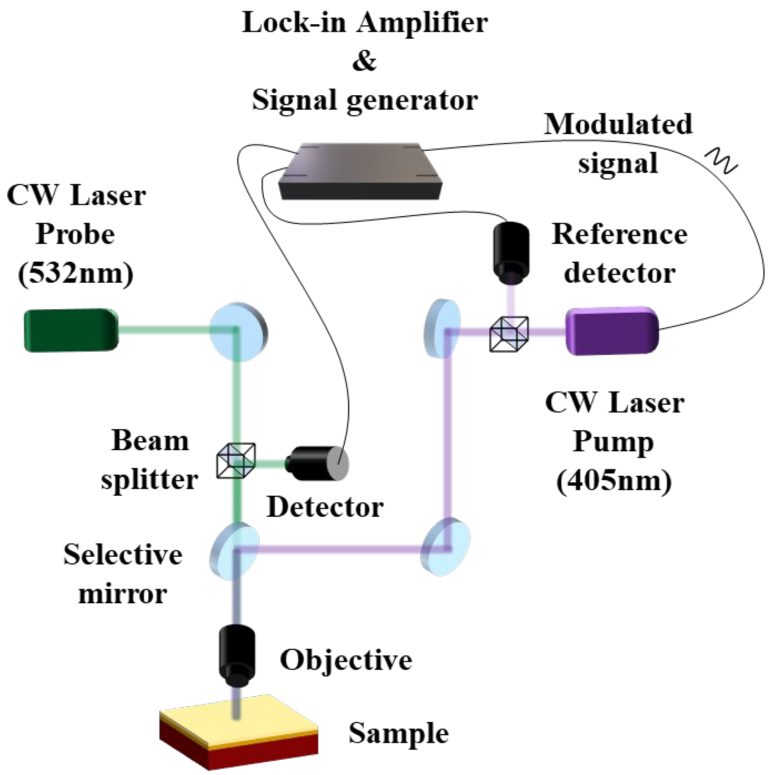

Figure S3. Schematic illustration of frequency-domain thermoreflectance (FDTR) setup.

Figure S4.

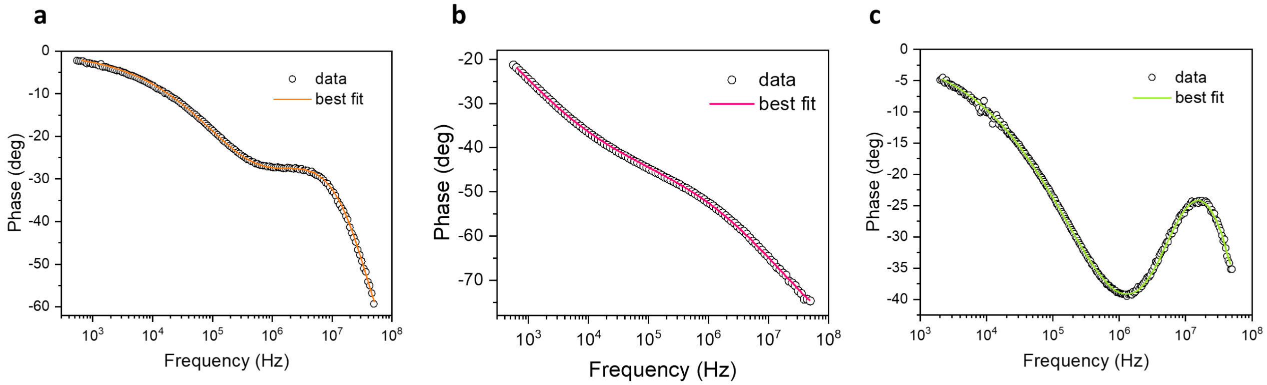

Figure S4. FDTR data plots with best fits of a, Silicon, **b,** Quartz, **c,** Copper.

Figure S5.

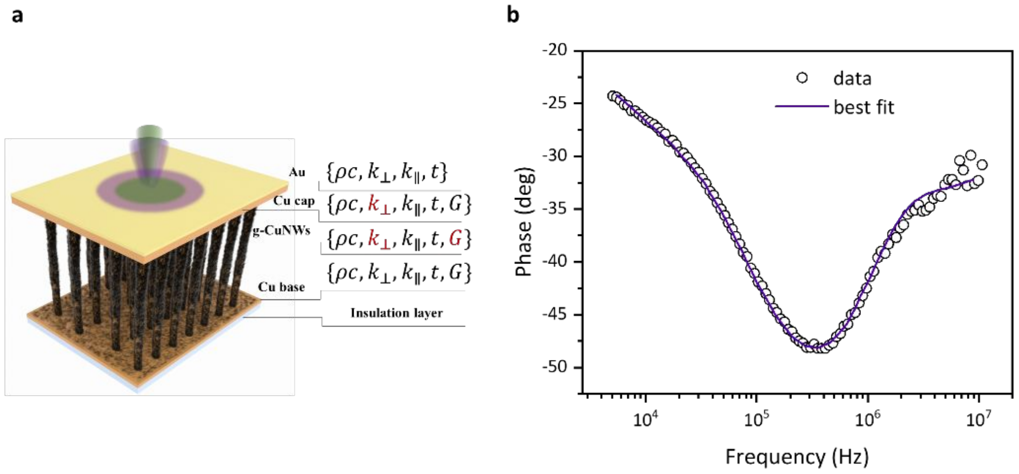

Figure S5. a, Configuration of the measurement of the 3D "sandwich" structure, in which the parameters in red are to be fitted. **b**, Experimental data and best fit

Figure S6.

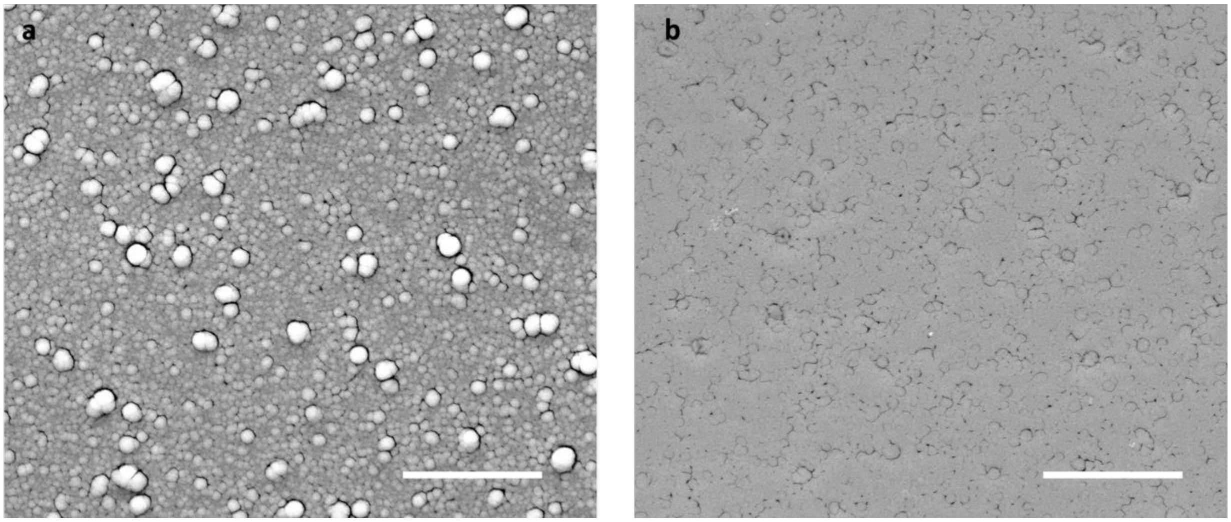

Figure S6. SEM top-view images of a, original Cu base layer, **b,** Cu base layer after ion milling. Scale bar: 10 μm

Figure S7.

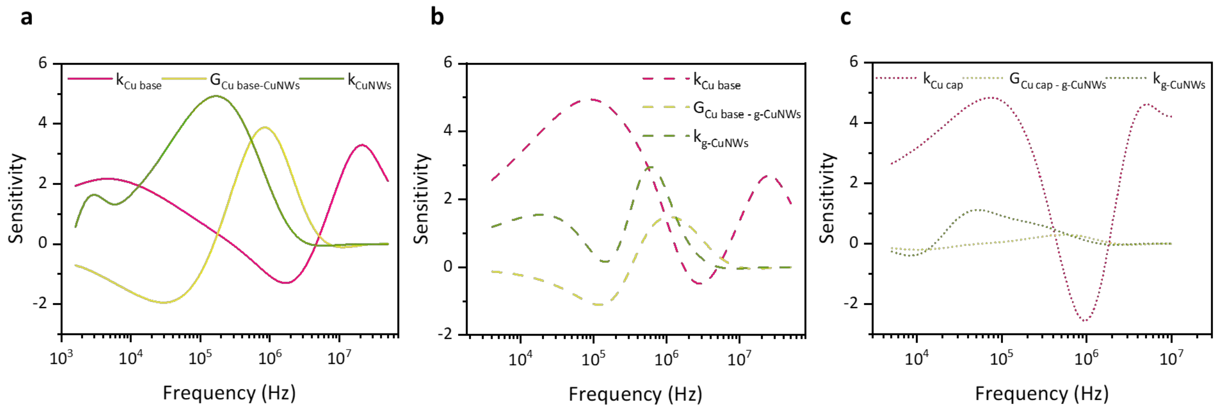

Figure S7. FDTR fitting sensitivity analysis of a, Au/Cu base/CuNWs/insulation layer, **b,** Au/Cu base/g-CuNWs/insulation layer, **c,** Au/Cu cap/g-CuNWs/Cu base/insulation layer configurations.

Figure S8.

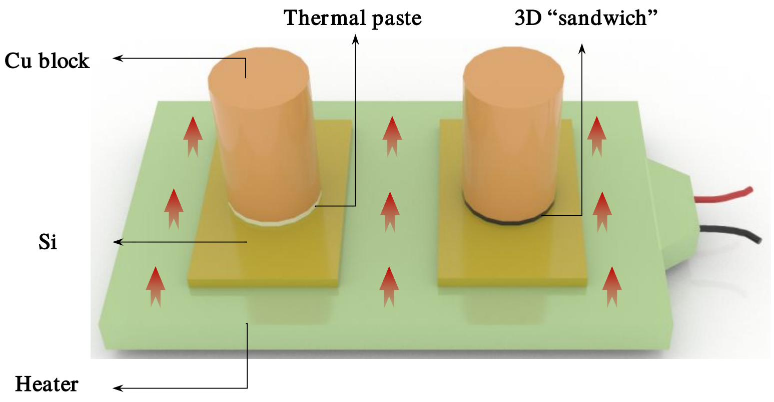

Figure S8. Schematic of experimental setup for infrared thermal mapping.

Figure S9.

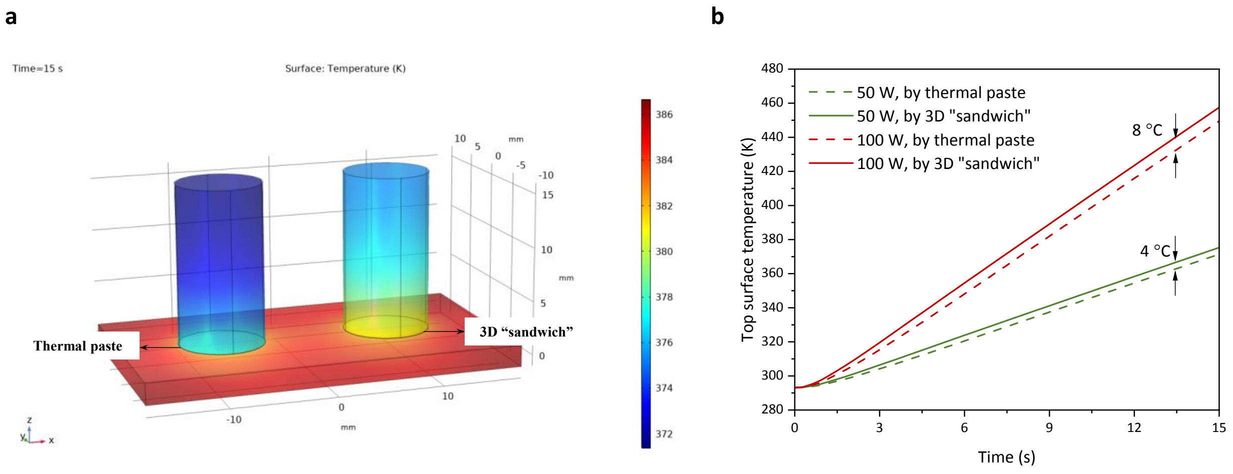

Figure S9. Time dependent COMSOL simulation of thermal mapping. a, Temperature distribution after 15 s. **b,** Top surface temperature versus time at different power supplies.

Figure S10.

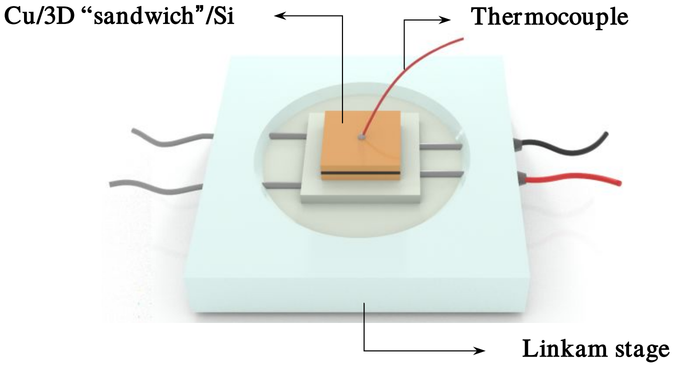

Figure S10. Schematic of experimental setup for temperature cycling test.

Figure S11.

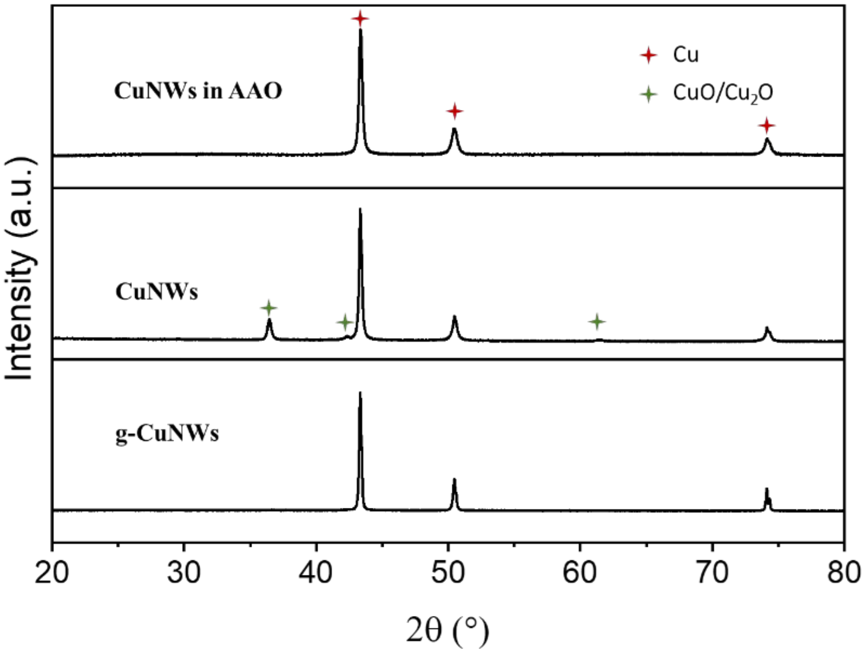

Figure S11. X-Ray Diffraction patterns of CuNWs in AAO, CuNWs, and g-CuNWs. The copper oxides signals occur on CuNWs after wet etching and are eliminated by the PECVD process.

Table S1.

Table S1. Summary of Young's modulus and shear modulus data with standard deviation from multiple measurements

	Young's modulus (MPa)	Shear modulus (MPa)
CuNWs	$0.123 \pm 5.73e\text{-}3$	$0.447 \pm 2.75e\text{-}1$
g-CuNWs	$0.145 \pm 1.73e\text{-}2$	$0.738 \pm 2.36e\text{-}1$
3D "sandwich"	$1.350 \pm 2.59e\text{-}2$	$1.044 \pm 2.89e\text{-}1$

Table S2.

Table S2. Summary of fitted results based on CuNWs, g-CuNWs, and 3D "sandwich" thermal measurements

	Cu base *k* (W/m K)	Cu cap *k* (W/m K)	Cu base-nanowire boundary conductance G (W/m^2K)	Cu cap-nanowire boundary conductance G (W/m^2K)	Nanowires *k* (W/m K)	Total resistance (mm^2 K/W)
CuNWs/Cu base	315 ± 44		3.1e7 ± 2.5e6		65 ± 6	
Cu cap/g-CuNWs/Cu base #1	355 ± 53	359 ± 50	2.9e7 ± 2.3e6	9.5e8 ± 1.9e8	97 ± 14	0.27± 0.19
Cu cap/g-CuNWs/Cu base #2	386 ± 35	374 ± 52	2.1e7 ± 1.9e6	1.4e8 ± 2.9e7	112 ± 16	0.26± 0.17
Cu cap/g-CuNWs/Cu base #3	357 ± 32	294 ± 44	2.4e7 ± 2.1e6	9.5e7 ± 7.6e6	128 ± 19	0.24± 0.13

References

(1) Marques, V. M. F.; Johnston, C.; Grant, P. S. Nanomechanical Characterization of Sn–Ag–Cu/Cu Joints—Part 1: Young's Modulus, Hardness and Deformation Mechanisms as a Function of Temperature. *Acta Materialia* **2013**, *61*, 2460–2470.

(2) Pharr, G. M.; Herbert, E. G.; Gao, Y. The Indentation Size Effect: A Critical Examination of Experimental Observations and Mechanistic Interpretations. *Annu. Rev. Mater. Res.* **2010**, *40*, 271–292.

(3) Gong, W.; Li, P.; Zhang, Y.; Feng, X.; Major, J.; DeVoto, D.; Paret, P.; King, C.; Narumanchi, S.; Shen, S. Ultracompliant Heterogeneous Copper–Tin Nanowire Arrays Making a Supersolder. *Nano Lett.* **2018**, *18*, 3586–3592.

(4) Schmidt, A. J. Optical Characterization of Thermal Transport from the Nanoscale to the Macroscale, Massachusetts Institute of Technology, 2008.

(5) Cahill, D. G. Analysis of Heat Flow in Layered Structures for Time-Domain Thermoreflectance. *Review of Scientific Instruments* **2004**, *75*, 5119–5122.

(6) Malen, J. A.; Baheti, K.; Tong, T.; Zhao, Y.; Hudgings, J. A.; Majumdar, A. Optical Measurement of Thermal Conductivity Using Fiber Aligned Frequency Domain Thermoreflectance. *Journal of Heat Transfer* **2011**, *133*, 081601.

(7) Ong, W.-L.; Rupich, S. M.; Talapin, D. V.; McGaughey, A. J. H.; Malen, J. A. Surface Chemistry Mediates Thermal Transport in Three-Dimensional Nanocrystal Arrays. *Nature Mater* **2013**, *12*, 410–415.

(8) Ong, W.-L.; O'Brien, E. S.; Dougherty, P. S. M.; Paley, D. W.; Fred Higgs III, C.; McGaughey, A. J. H.; Malen, J. A.; Roy, X. Orientational Order Controls Crystalline and Amorphous Thermal Transport in Superatomic Crystals. *Nature Mater* **2017**, *16*, 83–88.
